# Grey and white matter alteration in patients with patent foramen ovale: A cross-sectional observational study using combined surface-based morphometry and voxel-based morphometry

**DOI:** 10.1097/MD.0000000000050064

**Published:** 2026-08-14

**Authors:** Ying Yang, Yong Ye, Qi Li, Zaiqiang Zhang, Huibo Wang

**Affiliations:** a Department of Cardiology, The First College of Clinical Medical Science, China Three Gorges University and Yichang Central People’s Hospital, Yichang, Hubei, China; b Institute of Cardiovascular Diseases, China Three Gorges University, Yichang, Hubei, China; c Department of Cardiology, Hubei Key Laboratory of Ischemic Cardiovascular Disease, Yichang, Hubei, China; d Department of Cardiology, Hubei Provincial Clinical Research Center for Ischemic Cardiovascular Disease, Yichang, Hubei, China; e Department of Radiology, The First College of Clinical Medical Science, China Three Gorges University and Yichang Central People’s Hospital, Yichang, Hubei, China.

**Keywords:** magnetic resonance imaging, migraine, patent foramen ovale, surface-based morphometry, voxel-based morphometry

## Abstract

Patent foramen ovale (PFO) is closely associated with migraine. However, the mechanism remains unclear. Surface-based morphometry (SBM) and voxel-based morphometry (VBM) were widely used to study microstructural changes in the brain. We aimed to use SBM and VBM to investigate microstructural changes in the brain in migraineurs with PFO. A total of 20 migraineurs were enrolled in the PFO group, and 20 TCD-negative migraineurs, matched by gender and similar age, were included in the control group. All patients underwent brain MRI. 3D-T1-WI imaging was used to reconstruct and post-process for VBM and SBM analysis. There were no significant differences in their baseline clinical characteristics. VBM results showed that white matter (WM) volumes decreased in six relevant brain regions in the PFO group: cerebellar vermis VIII, right caudate, left superior temporal gyrus, left fusiform, left posterior cingulate gyrus, and left postcentral gyrus (*P* < .05 or *P* < .01). Also, the gray matter (GM) volumes of the bilateral hippocampus were significantly decreased in the PFO group (*P* < .01). Furthermore, SBM results showed that the cortex of the right superior frontal gyrus was thicker, the sulcus of the right rostral middle frontal gyrus was deeper, and the fractal dimension of the left superior parietal gyrus was more complex in the PFO group, but these results couldn’t pass FDR correction (*P* > .05). This cross-sectional study demonstrates that migraine patients with PFO exhibit focal gray and white matter volume reductions in multiple pain, cognition, and emotion-related brain regions. These structural alterations may represent important neural substrates underlying PFO-related migraine.

## 1. Introduction

Patent foramen ovale (PFO) is a congenital heart disease present in about 25% of adults, with an incidence of 20.2% in people over 80 years, 25% in those aged 30 to 79 years, and 30% in those aged 1 to 29 years.^[1]^ The foramen ovale is a physiological channel through which the atrial septum forms during embryonic development. If the channel does not close completely after age 3, it is called PFO.^[2]^ Generally, the pressure in the left atrium is higher than that in the right atrium and the pulmonary vein, which will not result in a right-to-left shunt or a small shunt. However, when right atrial pressure increases in PFO patients, significant right-to-left shunting occurs, leading to microthrombi and unmetabolized headache factors in the venous system entering the arterial system through the unclosed foramen ovale, ultimately leading to abnormal embolism and migraine.^[3]^

Migraine, stroke, and peripheral arterial embolism are the major complications of PFO. Additionally, PFO is an independent risk factor for migraine.^[4]^Notably, the detection rate of PFO in migraineurs was 3.19 times higher than in healthy controls, and the incidence rate of PFO in migraineurs with aura was 2.32 times higher than in migraineurs without aura.^[5]^ However, the mechanism by which PFO causes migraine is unclear, mainly including the microembolus mechanism and the headache factor shunt mechanism.^[6,7]^ A recent study showed that closing the unclosed foramen ovale can significantly improve symptoms, including migraines.^[8]^ PFO closure might relieve migraine by improving 5-HT clearance metabolism and ameliorating redox reactions.^[9]^ Therefore, focusing on brain structure and function changes in PFO patients is crucial to understanding the mechanism of migraine caused by PFO, so as to guide treatment.

Voxel-based morphometry (VBM) can accurately measure the volume of total intracranial volume (TIV), gray matter (GM), white matter (WM), and cerebrospinal fluid (CSF) volumes and identify subtle structural changes in the patient’s brain.^[10]^ Surface-based morphometry (SBM) can reconstruct a 3-dimensional model of the cerebral cortex by measuring each vertex of brain tissue, reproducing the complex cortical topology of patients and obtaining cortical data such as cortical thickness (CT), sulcal depth (SD), gyrification index (GI), and fractal dimension (FD).^[11]^ VBM and SBM have been widely used to study micro changes in brain structure, which can accurately depict the cranial morphological structure.^[12,13]^ Compared with the control group, there were significant differences in morphological indicators such as GM volume and CT in related brain areas in migraineurs.^[14,15]^ The small changes in brain structure in migraineurs were mainly concentrated in brain areas such as the thalamus, brainstem, and trigeminal pathway, which are closely related to migraine.^[16,17]^ Although some researchers have studied changes in brain morphology and structure in migraineurs using VBM and SBM, there is a lack of research on changes in brain microstructure in migraineurs with PFO. Therefore, our study aims to investigate the microstructural changes in the brain of migraineurs with PFO using VBM and SBM techniques, providing potential imaging evidence for the mechanism of migraine caused by PFO, so as to guide early clinical intervention, diagnosis, and treatment of PFO.

## 2. Materials and methods

### 2.1. Participants

**Figure 1.**
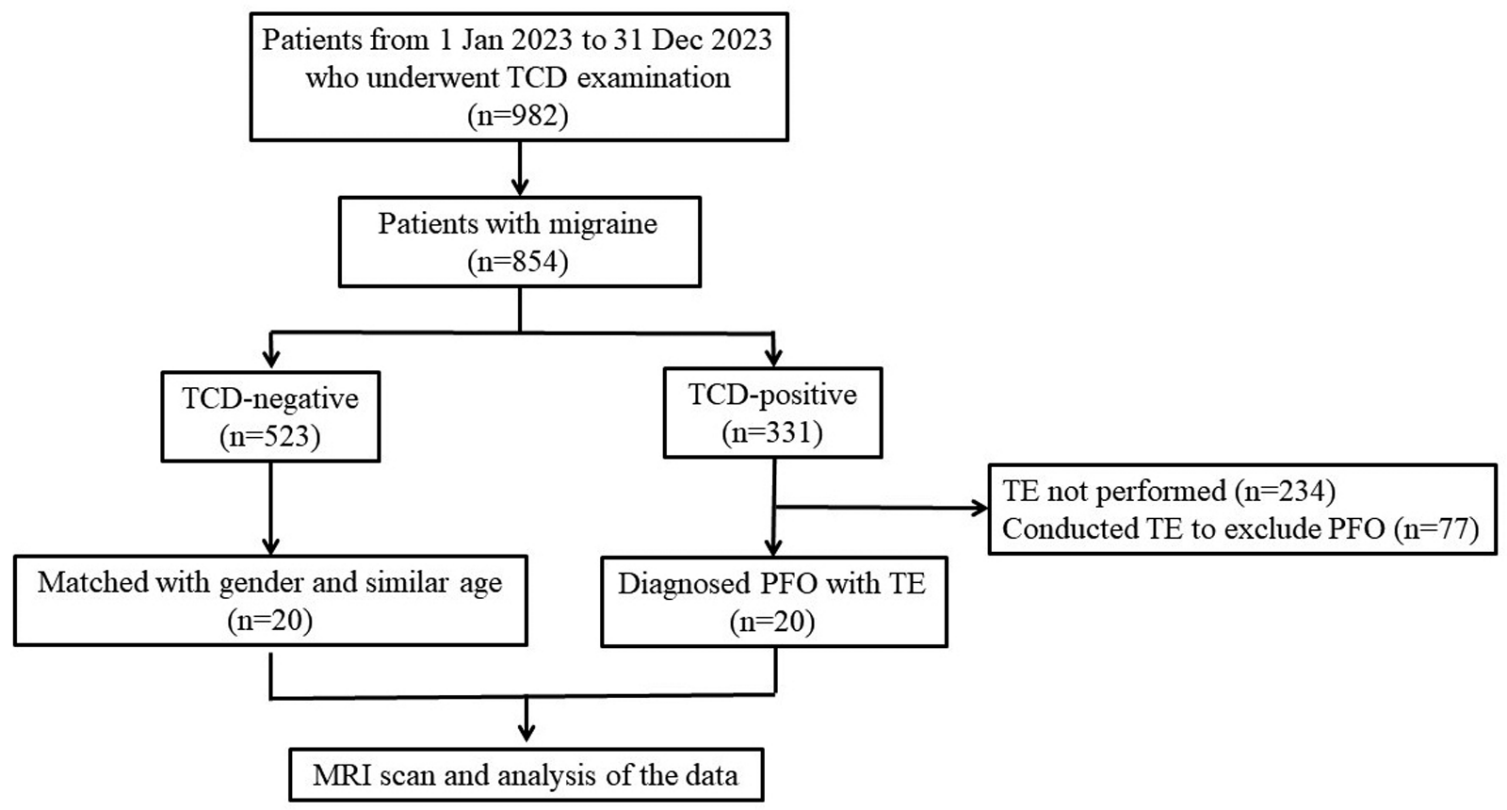
Flowchart of patients’ enrollment. MRI = magnetic resonance imaging, PFO = patent foramen ovale, TCD = transcranial Doppler bubble test, TE = transesophageal echocardiography.

Migraine diagnosis was established by experienced neurologists in strict accordance with the International Classification of Headache Disorders, third edition (ICHD-3) criteria. Migraineurs who underwent transcranial Doppler bubble test (TCD) in Yichang Central People’s Hospital from January 1, 2023 to December 31, 2023 were collected. The flowchart of patients’ enrollment was shown in Figure 1. Migraineurs with a positive TCD result and confirmed PFO by transesophageal echocardiography (TEE) were included in the PFO group. In total, 20 patients were enrolled in the PFO group, and 20 TCD-negative migraineurs matched by gender and similar age (±3 years) were included in the control group. This study was approved by the ethics committee of Yichang Central People’s Hospital (No. 2024-159-01). All participants in this study were informed of the examination procedures and precautions prior to the MRI examination and signed an informed consent form.

Inclusion criteria: Migraineurs who have undergone TCD. Exclusion criteria were as follows: after closure of PFO; other congenital or acquired cardiac diseases such as atrial septal defect, ventricular septal defect, and arteriovenous malformations; acute infectious diseases; severe hepatic and renal dysfunction; acute cardiovascular diseases such as heart failure or atrial fibrillation; acute cerebrovascular diseases such as cerebral infarction or cerebral hemorrhage; those with intracranial space-occupying lesions or brain trauma; obvious functional brain diseases such as mental illness, emotional/sleep disorders, and severe brain atrophy; and MRI contraindications such as pacemakers, artificial metal valves, claustrophobia, and patients who cannot effectively complete the MRI scan.

### 2.2. Transcranial Doppler bubble test and transesophageal echocardiography

TEE and TCD are the two most commonly used diagnostic methods for PFO in clinical practice. Among them, TEE is the “gold standard” for diagnosing PFO, which can intuitively display the dynamic changes and surrounding structures of PFO and can directly measure the diameter and length of PFO in a resting state and during the Valsalva maneuver. TCD uses shaking physiological saline to form microbubbles, which flow back to the right atrium, right ventricle, and pulmonary artery with the blood. As microbubbles cannot pass through the pulmonary capillary network, bright and dense bubbles can be seen in the right atrium under normal conditions, while there is no bubble development in the left atrium. When the patient has PFO and the right atrial pressure exceeds the left atrial pressure, microbubbles enter the left atrium, causing bubble imaging in the left atrium. When patients have pulmonary arteriovenous shunting or an atrial septal defect, TCD may also be positive, so PFO patients need to undergo TEE examination to further confirm PFO.

### 2.3. MRI data acquisition

All patients underwent brain MRI within 1 week of the diagnosis of PFO using the 3.0T MRI (Ingenia CX, Philips Healthcare, Netherlands), and the coil was a dedicated 32-channel brain coil. We removed metal objects from the patient before the MRI scan and informed the patient of the precautions to be taken during the scan. The sequences collected mainly included conventional brain MRI sequences (T1-WI, T2-WI, T2-FLAIR-WI) and 3D-T1-WI sequences. Conventional sequences can exclude patients with intracranial lesions, 3D-T1-WI provided brain structural imaging data, and the main parameters were as follows: sagittal scans, 192 slices; TR = 1600 ms; TE = 2.13 ms; flip angle = 12°; matrix = 224 × 256; 1 mm isotropic voxels; field of view: 256 mm × 256 mm × 211 mm; scanning time 6 minutes 12 seconds.

### 2.4. Image quality control and assessor information

All MRI images were evaluated by 2 experienced radiologists (with more than 5 years of neuroimaging experience) who were blinded to group allocation (PFO group or control group). Standard image quality control criteria were applied: No severe motion artifacts, ghosting, or signal inhomogeneity; complete brain coverage without truncation or field bias; and satisfactory contrast and signal-to-noise ratio for reliable segmentation. Scans failing quality control were excluded before further processing. All structural imaging processing (VBM and SBM) was performed by a trained rater blinded to clinical information using standardized automated pipelines to ensure consistency.

### 2.5. Statistical analysis

#### 2.5.1. VBM analysis

The structural data processing software mainly included MRIcron, MATLAB R2022b, SPM 12, and CAT12. Data processing mainly included conversion of the original data format, registration, segmentation, smoothing, and statistical analysis. The original data format was DICOM, which was converted to NIfTI format by the MRIcron software. The SPM12 and CAT12 software packages were loaded into the MATLAB R2022b platform, the converted data were matched to the Montreal Neurological Institute standard spatial template for registration, and the GM, WM, and CSF volume data were automatically segmented. Select the segmentation option in CAT12, enable modulation, use DARTEL registration, and smooth with a kernel of 8 mm. Considering the relatively small sample size of this study, only total intracranial volume (TIV) was included as a covariate in VBM and SBM analyses to avoid overfitting and loss of statistical power. Age, sex, migraine duration, and aura status were well balanced between groups (all *P* > .05) in the baseline matching process. Then, the 2-sample *t* test and Gaussian random field theory were used for FDR correction in SPM 12 with a threshold of *P* < .05 and cluster size *k* > 30.

#### 2.5.2. SBM analysis

The data processing of SBM was the same as that of VBM, based on the MATLAB R2022b data processing platform, employing SPM12 and CAT 12 software packages, and performing registration, segmentation, reconstruction, and smoothing of NIfTI format data regarding the Montreal Neurological Institute standard space. CAT12 was also used for image quality control and consistency detection, and the unqualified files were excluded. The post-segmentation CT data were processed with FWHM 15 mm Gaussian kernel smoothing, and the SD, GI, and FD data were processed with FWHM 20 mm Gaussian kernel smoothing. Finally, statistical analysis was performed in CAT12, with *P* < .05, and FDR correction was applied. Only results that survived FDR correction were considered statistically significant; uncorrected results were reported as exploratory and descriptive only.

### 2.6. Other statistical analyses

SPSS 19.0 software (IBM SPSS Statistics for Windows, Version 19.0., IBM Corp, Armonk) was used for statistical analysis. Measurement data were expressed as mean ± standard deviation, and a 2 independent samples *t* test was used to compare between the 2 groups. Counting data were expressed as frequency (percentage), and the chi-squared test or Fisher exact probability method was used to compare between 2 groups. Pearson correlation analysis was used to analyze the correlation between the GM volume, WM volume, CT, SD, GI, FD of significant brain regions, and PFO length and PFO diameter. *P* < .05 was considered statistically significant.

## 3. Results

### 3.1. Patient enrollment and baseline clinical characteristics

A total of 40 patients were included in this study, including 20 patients in the PFO group, with 17 females, a mean age of 39.30 ± 15.72 years, and 20 patients in the control group, with 18 females, and a mean age of 37.25 ± 10.07 years. The baseline clinical characteristics of the 2 groups were shown in Table 1. There were no significant differences between the PFO and control patients regarding age, sex, hypertension, diabetes mellitus, hyperlipidemia, migraine duration, and the presence of aura (all *P* > .05). PFO length and diameter detected by TE were also shown in Table 1.

**Table 1:** Study participants’ baseline clinical characteristics.

Item	PFO (N = 20)	Control (N = 20)	*t*/χ^2^/Z	*P* value
Age (yr, $\bar{x}\pm s$)	39.30 ± 15.72	37.25 ± 10.07	*t* = 0.461	.650
Female (n, %)	17 (85)	18 (90)	χ^2^ = 0.229	.633
Hypertension (n, %)	2 (10)	1 (5)	**–** *	.548
Diabetes mellitus (n, %)	1 (5)	1 (5)	**–** *	1.000
Hyperlipidemia (n, %)	3 (15)	1 (95)	**–** *	.292
Time of migraine (mo) M (P25, P75)	13.04 (6.52, 130.36)	13.04 (0.13, 65.18)	*Z* = −0.154	.878
The presence of aura (n, %)	8 (40.00)	10 (50.00)	0.404	.525
PFO length (mm, $\bar{x}\pm s$)	12.17 ± 3.85	**–**	**–**	**–**
PFO diameter (mm, $\bar{x}\pm s$)	1.55 ± 0.30	**–**	**–**	**–**

PFO = patent foramen ovale.

* Using Fisher exact probability method.

### 3.2. VBM results

#### 3.2.1. Comparison of TIV, CSF, and total GM, WM volumes

As shown in Table 2, there were no significant differences in TIV, CSF, and total GM and WM volumes between the PFO and control groups.

**Table 2:** Comparison of TIV, CSF, and total GM, WM volumes between the 2 groups.

	PFO (N = 20)	Control (N = 20)	*t*	*P* value
TIV (cm^3^)	1433.86 ± 127.32	1424.57 ± 91.43	0.28	.78
CSF (cm^3^)	285.48 ± 69.49	265.34 ± 61.62	1.03	.31
Total GM (cm^3^)	651.23 ± 67.79	650.34 ± 50.56	0.05	.96
Total WM (cm^3^)	497.15 ± 61.05	508.89 ± 31.50	−0.82	.42

CSF = cerebrospinal fluid, GM = gray matter, PFO = patent foramen ovale, TIV = total intracranial volume, WM = white matter.

#### 3.2.2. Comparison of WM and GM volumes of the relevant brain regions

**Figure 2.**
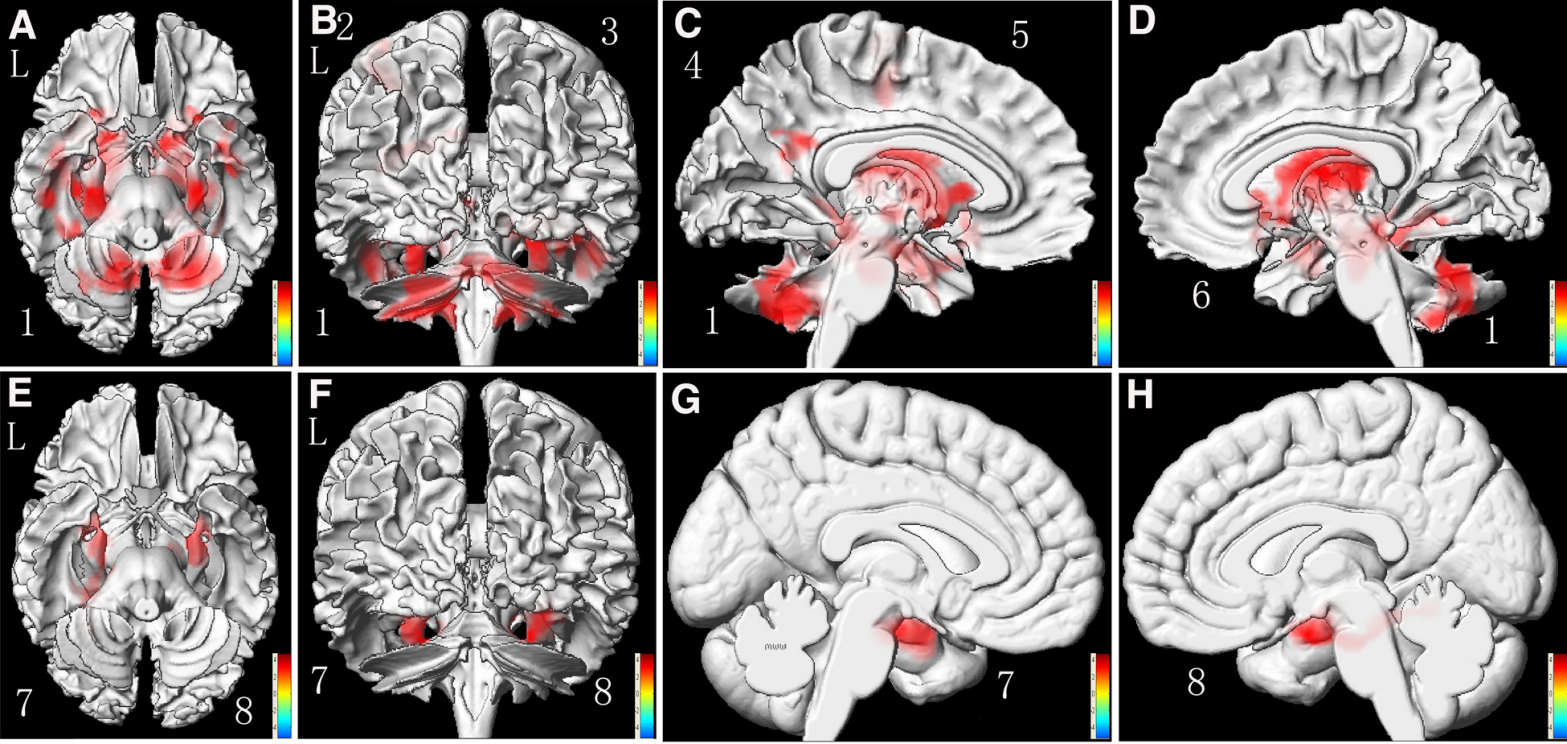
All brain regions with significant differences in WM volumes (A–D), GM volumes (E–H); 1: Bilateral vermis-VIII; 2: Left postcentral gyrus; 3: Left superior temporal gyrus; 4: Left fusiform; 5: Left posterior cingulate gyrus; 6: Right caudate; 7: Left hippocampus; 8: Right hippocampus. WM = white matter.

Compared with the control group, WM volumes decreased in 6 relevant brain regions in the PFO group, including cerebellar vermis VIII, right caudate, left superior temporal gyrus, left fusiform, left posterior cingulate gyrus, and left postcentral gyrus (*P* < .05 or *P* < .01). The GM volumes of the bilateral hippocampus were significantly decreased in the PFO group compared to those in the control group (all *P* < .01). However, there were no increased WM or GM volumes in any brain region in the PFO group, as shown in Table 3 and Figure 2.

**Table 3:** Comparison of WM, GM volumes of the relevant brain regions between the 2 groups.

Anatomical description	MNI coordinates			Cluster size	*Z* Score	*P* (FDR correction)
	*X*	*Y*	*Z*			
WM volumes						
Bilateral vermis-VIII	4.5	−63	−27	5520	4.84	<.01*
Right caudate	15	−3	18	14,519	5.5	<.01*
Left superior temporal gyrus	−42	12	−21	924	4.26	<.05*
Left fusiform	−28.5	−27	−28.5	1958	6.60	<.01*
Left posterior cingulate gyrus	−9	−52.5	28.5	803	5.93	<.05*
Left postcentral gyrus	−39	−19.5	52.5	1008	5.34	<.05*
GM volumes						
Left hippocampus	−27	−10.5	−21	1445	5.23	<.01*
Right hippocampus	25.5	−9	−19.5	1996	4.45	<.01*

*X*, *Y*, and *Z* correspond to the coordinates of the peak-activated voxel.

FDR = false discovery rate, GM = gray matter, MNI = Montreal Neurological Institute, PFO = patent foramen ovale, WM = white matter.

* PFO < control.

Correlation analysis showed no significant correlations between the WM volumes of the cerebellar vermis VIII, right caudate, left superior temporal gyrus, left fusiform, left posterior cingulate gyrus, left postcentral gyrus, and the anatomical parameters of foramen ovale (length and diameter), nor between the GM volumes of the bilateral hippocampus and the anatomical parameters of foramen ovale (length and diameter; all *P* > .05).

### 3.3. SBM results

Exploratory SBM analysis showed trends of differences in cortical thickness (CT), sulcal depth (SD), and fractal dimension (FD) between groups (uncorrected *P* < .05). However, none of these differences survived FDR correction (all *P* > .05). These observations should be considered preliminary and exploratory without statistical significance. Specifically, trends toward a thicker right superior frontal cortex, deeper right rostral middle frontal sulcus, and more complex left superior parietal gyrification were observed in the PFO group. The results were shown in Table 4.

**Table 4:** Comparison of CT, SD, and FD between the 2 groups

	Anatomical description	MNI coordinates			Cluster size	*Z* score	*P* value	*P* (FDR correction)
		*X*	*Y*	*Z*				
CT	Right superior frontal gyrus	17	2	65	35	3.17	<.01*	.774
SD	Right rostral middle frontal gyrus	29	50	−30	62	3.57	<.01*	.368
FD	Left superior parietal gyrus	−28	−57	65	38	3.4	<.01*	.791
FD	Left supramarginal gyrus	−64	−32	28	32	3.16	<.01†	.801

*X*, *Y*, and *Z* correspond to the coordinates of the peak-activated voxel.

CT = cortical thickness, FD = fractal dimension, FDR = false discovery rate, MNI = Montreal Neurological Institute, PFO = patent foramen ovale, SD = sulcal depth.

* PFO < control.

† PFO > control.

## 4. Discussion

In this study, we used 2 morphometric methods, VBM and SBM, for the first time to investigate brain microstructural changes in migraineurs with PFO. Results showed that the volumes of the cerebellar vermis VIII, right caudate, left superior temporal gyrus, left fusiform, left posterior cingulate gyrus, left postcentral gyrus, and bilateral hippocampus were decreased in PFO patients. SBM results showed that the cortex of the right superior frontal gyrus was thicker, the sulcus of the right rostral middle frontal gyrus was deeper, and the GI of the left superior parietal gyrus was more complex in the PFO group, but these results couldn’t pass FDR correction.

VBM has been widely used to study brain structure and function in migraine patients, and functional and structural changes in cortical and subcortical pain-related brain regions were closely associated with migraine.^[15,18]^ One study found that the fractional anisotropy of cerebellar vermis VI was significantly reduced in migraineurs without aura.^[19]^ Our study found that the WM volume of cerebellar vermis VIII was significantly reduced. The GM volume of the caudate nucleus increased in migraineurs, and the connection between the caudate nucleus and the cingulate gyrus was significantly increased.^[20,21]^ Our study found that the WM volume of the caudate and cingulate gyrus was significantly reduced in the PFO group. Other studies have shown that the microstructure of the fusiform gyrus and postcentral gyrus was also significantly altered in PFO patients.^[22,23]^ Notably, the alteration of GM structure in the temporal gyrus, especially the hippocampus, was a key pathological feature in migraineurs without aura.^[24,25]^ Patients with chronic pain had significant reductions in the anterior cingulate and left hippocampus, consistent with the results of our study. In summary, there were significant changes in the microstructure of brain regions in migraineurs, including the hippocampus, cerebellar vermis VIII, right caudate, left superior temporal gyrus, left fusiform, left posterior cingulate gyrus, and left postcentral gyrus.

SBM is an essential technical tool for the study of the cortex of the central nervous system, reflecting the complex topological structure of the cerebral cortex through 3-dimensional reconstruction of the cerebral cortex structure. Research has shown that the surface area of the frontal cortex was significantly reduced in migraineurs with visual, somatosensory, and language impairments.^[26]^ Compared with the normal control group, CT in the temporal, frontal, insular, postcentral, primary, and associative visual areas was significantly thinner in patients with migraine with aura, suggesting that migraine with aura may be associated with cortical thinning in multiple cortical regions.^[27]^ In contrast, another study of migraine with aura demonstrated that there were no significant differences in CT, GI, and SD between normal healthy controls and migraine with aura.^[28]^ There were also no significant differences in CT between the 2 groups in somatosensory, cingulate, or visual motor processing cortex.^[29]^ Moreover, a coordinate-based meta-analysis technique showed no significant consistency in CT among migraine patients.^[30]^ Exploratory SBM analysis revealed some morphological trends in frontal and parietal regions, but these did not reach statistical significance after FDR correction. Therefore, these findings are preliminary and exploratory and should be interpreted with caution.

Microemboli and headache factors that are not metabolized by pulmonary circulation can enter the cerebral circulation through an unclosed foramen ovale, leading to chronic ischemia or inflammatory reactions. The anatomical characteristics (diameter and length) of the unclosed foramen ovale may affect the probability of microemboli and headache factors passing through and the degree of microstructural damage to the brain.^[31,32]^ Research has found that a larger diameter PFO (>4 mm) is associated with more severe white matter lesions, possibly due to more microembolic events, while a longer PFO may affect the blood flow pattern of right to left shunting and be associated with gray matter and white matter atrophy, suggesting a possible risk of chronic hypoxia.^[33,34]^ Other studies have shown that the right to left shunt volume (related to PFO diameter, length, and shunt volume) is significantly correlated with a decrease in hippocampal and thalamic gray matter volume, suggesting that hemodynamic disorders may drive gray matter atrophy.^[35]^ This study found that there is no significant correlation between the white matter volume and gray matter volume of PFO patients and the anatomical parameters of the foramen ovale (length and diameter). It may be related to measurement method errors, population heterogeneity (such as differences in comorbidities), complexity of biological mechanisms (such as nonlinear effects or compensatory mechanisms), and statistical limitations (such as insufficient sample size or uncorrected multiple comparisons), which need to be further validated through imaging technology optimization and in-depth research on mechanisms.

VBM and SBM are brain structure imaging analysis methods developed alongside magnetic resonance neuroimaging technology in recent years, which are the most important technical means for quantitatively evaluating brain structure and function. Likewise, they are significant for studying the pathophysiological mechanisms and differential diagnosis of various cranial diseases. However, there are also many limitations and influencing factors, such as differences in study objects (subtype, disease course changes, treatment measures), scanning equipment, imaging sequences and parameters, and post-processing algorithms, which may cause inconsistent research results.

Though, we have found several microstructural changes in the brains of migraineurs with PFO, there are still some recognized limitations. First, this study only enrolled migraineurs with PFO and migraineurs without PFO, and no healthy control group was included. Therefore, we cannot definitively determine whether the observed brain structural differences are caused by PFO alone, migraine itself, or the combined effect of both. This constraint restricts the absolute clinical reference value of the main results to some extent. The main reason for not including healthy controls is that TEE, the gold standard for PFO diagnosis, is invasive, and healthy individuals are reluctant to undergo this examination, leading to difficulties in the accurate enrollment of healthy participants with or without PFO. Nevertheless, by comparing the 2 migraine groups, our design can minimize the confounding effect of migraine and highlight the potential additional impact of PFO on brain microstructure. Second, the influence of the clinical classification, the frequency and severity of migraine, medication use, and anatomical subtypes of PFO still need further analysis. Third, most studies have identified that the association between PFO and migraine with aura is more evident than that between PFO and migraine without aura; however, there were only 18 migraine patients with aura in this study, and we are currently collecting more migraine patients with aura to analyze the influence of PFO in migraine patients with aura. Fourth, this is a static transverse study to analyze the relationship between migraine and PFO; we will then dynamically observe the changes in brain microstructure after PFO closure surgery. Although age, sex, and headache characteristics were well matched between groups, these factors were not included as covariates in the statistical models due to the limited sample size, which may be a potential limitation. Future studies with larger sample sizes, multi-center design, and healthy control groups are warranted to clarify the independent effect of PFO on brain structure and improve the clinical reference value of relevant results.

## 5. Conclusion

We first used VBM and SBM to assess the microstructural changes in the brains of migraineurs with PFO. Results showed that the decreased volume of the vermis-VIII, right caudate, left superior temporal gyrus, left fusiform, left posterior cingulate gyrus, left postcentral gyrus, and bilateral hippocampus may be important damages from PFO in migraineurs.

## Acknowledgments

The authors thank AiMi Academic Services (www.aimieditor.com) for English language editing and review services.

## Author contributions

**Conceptualization:** Ying Yang, Yong Ye.

**Data curation:** Ying Yang, Yong Ye, Qi Li, Zaiqiang Zhang, Huibo Wang.

**Formal analysis:** Ying Yang, Qi Li.

**Funding acquisition:** Ying Yang, Yong Ye.

**Investigation:** Ying Yang, Qi Li, Huibo Wang.

**Methodology:** Ying Yang, Yong Ye, Qi Li, Huibo Wang.

**Resources:** Ying Yang, Qi Li, Zaiqiang Zhang.

**Software:** Yong Ye.

**Validation:** Ying Yang, Yong Ye, Qi Li.

**Visualization:** Yong Ye, Qi Li.

**Writing – original draft:** Ying Yang, Yong Ye, Qi Li.

**Writing – review & editing:** Ying Yang, Yong Ye, Huibo Wang.
